# Detection of Chronic Kidney Disease by Using Different Equations of Glomerular Filtration Rate in Patients with Type 2 Diabetes Mellitus: A Cross-Sectional Analysis

**DOI:** 10.7759/cureus.1352

**Published:** 2017-06-14

**Authors:** Sojib Bin Zaman

**Affiliations:** 1 Maternal and Child Health Division, International Centre for Diarrhoeal Disease Research, Bangladesh

**Keywords:** diabetes mellitus, egfr, thailand, ckd

## Abstract

Introduction

Chronic kidney disease (CKD) is a global threat due to its high mortality. It is essential to know the actual magnitude of diabetic CKD to design a specific management program. However, there is limited knowledge regarding the most suitable equation to measure CKD in patients with Type 2 diabetes mellitus (T2DM). This paper aimed to analyze estimated glomerular filtration rate (eGFR) based on different equations to detect the CKD among T2DM.

Methods

A hospital-based cross-sectional study was carried out, and a clinical registry was used to collect 4,042 T2DM patients from a large district hospital in Northeast Thailand. CKD patients were diagnosed when eGFR was less than 60 ml/min/1.73m^2^. Using Stata statistical software (StataCorp LP, College Station, TX), three standard equations, such as ‘modification of diet in renal disease (MDRD-4)’, ‘chronic kidney disease epidemiology collaboration (CKD-EPI)’, and ‘Cockcroft-Gault (C-G)' equations, were used to produce eGFR values to report and compare stages of CKD.

Results

The mean age of the patients was 61.4 (± 10.7) years and male to female ratio was 1:1.9. According to the MDRD-4, CKD-EPI, and C-G equation, the prevalence of diabetic CKD was 21.4%, 21.9%, and 31.4%, respectively, and the frequency of CKD Stage 3 to 5 was found to be different among T2DM. About 3,789 (93.9%) measurements appeared to be classified as different stages of CKD (Stages 1 to 5) between MDRD-4 and CKD-EPI equations (kappa: 0.905; 95% confidence interval (CI): 0.83 - 0.97, p < 0.001). However, this study found that the above-mentioned agreement was 70.9% between CKD-EPI and C-G equation (kappa: 0.56, 95% CI: 0.44 - 0.67, p < 0.001).

Conclusions

CKD-EPI equations can overcome the constraint of MDRD-4 and C-G equations to report CKD and can be used in patients with T2DM.

## Introduction

The prevalence of chronic kidney disease (CKD) has been estimated to be more than 13% globally [1-3]. According to statistics, approximately 200 million people suffer from CKD around the world today [4]. Diabetes mellitus is one of the leading causes of CKD around the world and responsible for causing high morbidity [5]. CKD might progress to renal failure, which can cause life-threatening complications for diabetic patients [6]. Dialysis, hemofiltration, and kidney transplantation are the available treatment options for the patients with CKD. However, this treatment protocol cannot reverse healthy kidney function back to normal but rather delays the progression of kidney damage [7]. CKD, as a complication of Type 2 diabetes mellitus (T2DM), has frequently been reported in Southeast Asian countries like Thailand [8]. Thailand is also geographically connected to the ‘stone belt’ zone where the renal stones are more common; this zone extends from the Central Asia to South Asia [9-10]. Therefore, kidney diseases due to T2DM or non-diabetic causes are common among the Thai population. However, the high burden of CKD due to T2DM poses a significant challenge to the Thai universal health coverage (UHC) system [11], which reiterates the importance of being aware of the recent diabetic CKD prevalence in Thailand.

Traditionally, serum creatinine concentration is used to diagnose CKD in many resource-poor settings [12]. However, the detection of CKD, depending on serum creatinine levels, might not be accurate when compared with other diagnostic approaches [13]. To illustrate, for older people and lean young females, this test is not a useful tool to detect renal disease [14]. Patients with reduced muscle mass may be related to reduced serum creatinine levels in their body [14-15]. Therefore, elderly patients might show lower serum creatinine concentrations despite having advanced kidney disease [13]. Direct measurement of the glomerular filtration rate (GFR) is reliable, but the procedure is cumbersome and expensive and hence, not very suitable in clinical settings. The alternate option is to use an estimated glomerular filtration rate (eGFR) rather than direct measurement [16]. Studies have used "modification of diet in renal disease - four variables (MDRD-4)", or "chronic kidney disease - epidemiology collaboration (CKD - EPI)", or "Cockcroft-Gault (C-G)" equations to report CKD [17]. Therefore, the reported CKD prevalence usually varies across the countries [18]. Some countries use "end-stage renal disease (ESRD)" to report the prevalence of CKD as well [19]. The exact estimation of the number of ESRD patients due to T2DM is crucial to know, as the ESRD patients are likely to utilize dialysis services or kidney transplantation in near future [19]. However, there are controversies in deciding which method is more accurate to report the prevalence of CKD among the diabetic patients. The aim of this study was to analyze eGFR in patients with T2DM and to evaluate different equations to detect CKD in patients with T2DM. 

## Materials and methods

### Study design and data source

The Institutional Review Board of Khon Kaen University (KKU) approved this study (approval #HE2247). This study performed a hospital-based cross-sectional study. Data was obtained from the clinical registry of diabetic patients who received medical care from a large district hospital in Northeast of Thailand. Based on the eligibility criteria, data was obtained from 4,042 T2DM patients between January 01, 2015 and December 31, 2015. A medical record number was used as a unique identifier to connect hospital records across the hospital’s electronic health record system. Pre-diagnosed diabetic patients who obtained a serum creatinine test in the hospital during the study period and were 18 years of age or older were included in the study. Patients with Type 1 or gestational diabetes mellitus were excluded from this study.

### Definition of CKD and its staging

This study used both conventional creatinine measurement and eGFR equations to determine CKD. A traditional cut-off point of serum creatinine (≥ 1.4 mg/dL if male and ≥ 1.2 mg/dL if female) was considered to detect CKD. In this study, CKD was defined based on the eGFR value below 60 mL/min/1.73 m2. The criteria were set according to the “National Kidney Foundation - Kidney Disease Outcomes Quality Initiative (NKF-KDOQI)” [17]. The staging of CKD was categorized according to NKF-KDOQI criteria based on different eGFR distribution: Stage 1: > 90 mL/min; Stage 2: 60 – 89 mL/min; Stage 3: 30 – 60 mL/min; Stage 4: 15-29 mL/min; and Stage 5: < 15 mL/min.

### Estimated eGFR equations

This study has used "modification of diet in renal disease - four variable (MDRD-4)", ‘‘chronic kidney disease - epidemiology collaboration (CKD - EPI)", and "Cockcroft-Gault (C-G)" equations to calculate eGFR to report CKD.

i. Four-variable MDRD equation [7]

 eGFR = 186.3 x S. Cr (mg/dL) - 1.154 x age (year) - 0.203 ( x 0.742 for women) (x 1.21 for non-Hispanic Black)    

           Here, S. Cr is serum creatinine in mg/dL

ii. CKD-EPI equation [17]

For females with S. Cr ≤ 62 µmol/L: eGFR = (144 + 22 if Black) x (Cr/0.7)^-0.329 x 0.993^age

For females with S. Cr > 62 µmol/L:  eGFR = (144 + 22 if Black) x (Cr/0.7)^-1.209 x 0.993^age

For males with S. Cr ≤ 80 µmol/L:     eGFR = (141 + 22 if Black) x (Cr/0.9)^-0.411 x 0.993^age

For males with S. Cr > 80 µmol/L:     eGFR = (141 + 22 if Black) x (Cr/0.9)^-1.209 x 0.993^age

            Here, the unit of S. Cr is in µmol/L

iii. Cockcroft-Gault equation [20]

eGFR = (140 - age) x weight x 1.04 (if female) / S. Cr, and

eGFR = (140 - age) x weight x 1.23 ( if male) / S. Cr

           Here, the unit of S. Cr is in µmol/L and unit of weight is by kilograms.

### Data management and statistical analysis

Data entry was performed by a group of skilled data operator under the supervision of a data management officer. Errors in data entry were revised after cross-checking both the laboratory records and clinical case recording forms. Frequencies and proportions were used to present categorical variables. Mean and standard deviation (SD) were considered to describe continuous variables. eGFR was estimated by using serum creatinine and additional covariates (age, sex, body weight, and non-Hispanic Black). Three standard equations (CKD-EPI, MDRD-4, and C-G) were used to produce eGFR values. Mann-Whitney U test was used to find out the comparison of different eGFR values. The kappa index was used to analyze the level of agreement to determine CKD stages, which were obtained by using three equations. However, in the absence of a gold standard method to estimate GFR, this study compared eGFR values obtained among the CKD-EPI, MDRD-4, and C-G equations. Stata, version 13 special edition (College Station, Texas, USA), was used for analyzing the data considering p-value < 0.05.

## Results

### Basic information about patient characteristics

The mean age of the T2DM patients was 61.4 (± 10.7) years, and the male to female ratio was 1:1.9. Respondents’ occupation were farmers (59.4%), different types of employment (28%), including day laborer, housewife, government employee, soldier, monk, etc., and 10.7% were unemployed. Only 1.3% participants were identified as current alcohol drinkers, and 3.8% of T2DM patients gave a positive history of current smoking. The mean serum triglycerides and low-density lipoprotein cholesterol (LDL-C) was 179 mg/dl and 110 mg/dl, respectively. Every diabetic patient was treated under the three schemes (the universal coverage scheme, Civil Service welfare, and Social Security scheme) of the Universal Health Coverage (UHC) who attended the participating hospital (Table 1).

**Table 1:** Basic Information About the Patient Characteristics (n = 4,042) Categorical data are presented as number (percentage); continuous data are shown as means ± SD. SD: standard deviation; LDL: low-density lipoprotein

Characteristics			Number	Percentage
Age				
Mean ± SD			61.4 ± 10.7	
Min : Max			20 - 95	
Sex				
Male			1379	34.2
Female			2,663	65.8
Universal Health Coverage (UHC)				
Universal coverage scheme			3,333	82.5
Civil Service welfare			486	12.0
Social Security scheme			223	5.5
Occupation				
Farmer			2,498	61.8
Different categories			1,110	27.5
Unemployment			434	10.7
Body Mass Index				
Mean ± SD			24.9 ± 4.0	
Min : Max			14.7 – 40.7	
Hypertension				
No			1,729	42.8
Yes			2,313	57.2
Serum Triglyceride				
Mean ± SD			179.18 ± 108.42	
Min : Max			24 - 947	
Glycated Hemoglobin (HbA1c)				
Mean ± SD			8.46 ± 2.19	
Min: Max			4.4 - 21	
LDL - Cholesterol				
Mean ± SD			110.14 ± 33.19	
Min : Max			78 - 578	
Current Smoker				
No			3,885	96.2
Yes			157	3.8
Current Alcohol Drinker				
No			3,986	98.6
Yes			56	1.4

### Prevalence of T2DM patients based on conventional method and eGFR equations

About 18.6% patients were categorized as CKD based on serum creatinine concentration. CKD was slightly higher among the males (19.4%) as compared to the females (18.2%) (Table 2).

**Table 2:** CKD According to Elevated Serum Creatinine Level CKD: chronic kidney disease

Serum Creatinine (mg/dl)	Number	Percentage
Male ≥ 1.4 (n = 1,379)	268	19.4
Female ≥ 1.2 (n = 2,663)	485	18.2
≥ 1.4 if Male and ≥ 1.2 if Female	753	18.6

**Figure 1:**
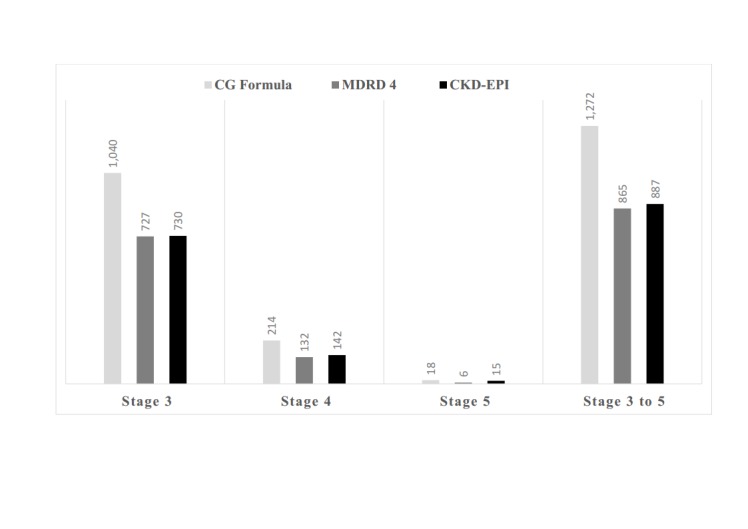
CKD distribution according to C-G, MDRD-4, and CKD-EPI equations CG: Cockcroft- Gault equation; CKD: chronic kidney disease; CKD-EPI: chronic kidney disease epidemiology; eGFR: estimated glomerular filtration rate; MDRD-4: modification of diet in renal disease (4 inputs, simplified)

According to the MDRD-4, CKD-EPI, and C-G equation, the mean value of eGFR was 83.9 mL/min/1.73m2, 78.7 mL/min/1.73 m2, and 75.3 mL/min/1.73 m2, respectively, among the T2DM patients (p < 0.05). About 21.9% patients were classified as CKD based on the CKD-EPI equation. The frequency of CKD Stages 3 to 5 was found to be dissimilar, considering the different equations. After the application of the MDRD-4 equation, the proportion of CKD was 21.4% (Table 3). The CKD-EPI equation driven CKD number was slightly higher in Stages 4 and 5 when compared with MDRD-4. However, the frequency of CKD was 1,272 (31.4%) according to the C-G equation. Patients with Stage 3 (n = 1,040) were much higher based on the C-G equation as compared to the MDRD-4 (n = 727) and the CKD-EPI equation (n = 730) (Figure 1).

However, the frequency of Stage 5 CKD patients was 0.37% and 0.15% considering the CKD-EPI and MDRD-4 equation, respectively, and it was found to be statistically significant (Table 3).

**Table 3:** Prevalence of CKD Based on Different eGFR Equation CKD: chronic kidney disease; eGFR: estimated glomerular filtration rate; MDRD-4: modification of diet in renal disease (4 inputs, simplified); CKD-EPI: chronic kidney disease epidemiology; CG: Cockcroft- Gault equation

CKD Stage	eGFR (mL/min/1.73 m^2^)	C-G Number (%)	MDRD-4 Number (%)	CKD-EPI Number (%)
1	> 90	1,634 (40.4)	1,997 (49.4)	2,021 (50.0)
2	60 – 89	1,136 (28.1)	1,180 (29.2)	1,134 (28.1)
3	30 – 60	1,040 (25.7)	727 (17.9)	730 (18.1)
4	15-29	214 (5.2)	132 (3.2)	142 (3.5)
5	< 15	18 (0.4)	6 (0.1)	15 (0.3)
CKD (3-5)	< 60 mL/min/1.73 m^2^	1,272 (31.4)	865 (21.4)	887 (21.9)

### Comparisons of different eGFR equations

Of the 4,042 measurements, 3,789 (93.9%) values were similarly classified as Stages 1 to 5 with MDRD-4 and CKD-EPI equations. The reported kappa index between the two equations, MDRD-4 and CKD-EPI, was 0.90 (95% confidence interval (CI): 0.83 - 0.97, p < 0.001) (Table 4). However, there was a 6.1% disagreement in classifying the CKD stages between two equations. About 28 patients were classified as Stages 4 and 5 using the CKD-EPI equation, but these patients actually were not the above-mentioned stages when we considered the MDRD-4 equation to detect the CKD stage.

**Table 4:** Comparisons of CKD Stages According to the CKD-EPI and MDRD-4 Equation Agreement: 93.9%; kappa: 0.905; 95% confidence interval (CI): 0.83 - 0.97, p < 0.001 CKD: chronic kidney disease; eGFR: estimated glomerular filtration rate; MDRD-4: modification of diet in renal disease (4 inputs, simplified); CKD-EPI:  chronic kidney disease epidemiology

eGFR according to MDRD-4	CKD Stage	eGFR according to CKD-EPI					
		1 (%)	2 (%)	3 (%)	4 (%)	5 (%)	Total (%)
	1	1,931 (47.8)	66 (1.7)	0	0	0	1,997 (49.4)
	2	90 (2.2)	1,049 (25.9)	41 (1.0)	0	0	1,180 (29.1)
	3	0	19 (0.5)	689 (17.1)	19 (0.5)	0	727 (18.0)
	4	0	0	0	123 (3.0)	9 (0.2)	132 (3.2)
	5	0	0	0	0	6 (0.1)	6 (0.2)
	Total	2,021 (50.0)	1,134 (28.1)	730 (18.1)	142 (3.5)	15 (0.3)	4,042 (100%)

However, this study found that agreement between the CKD-EPI and C-G equation was 2,869 (70.9%) with a kappa index of 0.56 (95% CI: 0.44 - 0.67, p < 0.001) (Table 5). Also, the agreement was found to be only 69.6% (kappa: 0.54, 95% CI: 0.44 - 0.67, p < 0.001) after comparing the MDRD-4 and C-G equation (data not shown).

**Table 5:** Comparisons of CKD Stages According to CKD-EPI and C-G Equation Agreement: 70.9%; kappa: 0.56; 95% confidence interval (CI): 0.44 - 0.67, p < 0.001 CKD: chronic kidney disease; eGFR: estimated glomerular filtration rate; MDRD-4: modification of diet in renal disease (4 inputs, simplified); CKD-EPI: chronic kidney disease epidemiology; C-G: Cockcroft-Gault equation

eGFR according to CKD-EPI	CKD Stage	eGFR according to C-G					
		1 (%)	2 (%)	3 (%)	4 (%)	5 (%)	Total (%)
	1	1,534 (37.9)	474 (11.7)	13 (0.3)	0	0	2,021 (50.0)
	2	80 (1.9)	623 (15.4)	431 (10.6)	0	0	1,134 (28.1)
	3	17 (0.4)	39 (0.9)	584 (14.4)	90 (2.2)	0	730 (18.1)
	4	0	0	14 (0.3)	119 (2.9)	9 (0.2)	142 (3.6)
	5	0	0	0	6 (0.1)	9 (0.2)	15 (0.4)
	Total	1,631 (40.4)	1,136 (28.1)	1,042 (25.8)	215 (5.3)	18 (0.4)	4,042 (100%)

## Discussion

This study found that 21.9% of T2DM patients were detected as CKD according to the CKD-EPI equation. However, the frequency of CKD was 31.4% and 21.4% based on the C-G and MDRD-4 equation, respectively. Therefore, MDRD-4 and CKD-EPI methods were coherent to report the CKD prevalence with a kappa index of 0.90. Based on the concentration of serum creatinine, the CKD was found to be 18.6% among the T2DM patients. Therefore, underestimation or overestimation of the prevalence of CKD due to the application of different detection methods has emerged as a future challenge for many developing countries [21]. However, the frequency of CKD in this study was found to be smaller in comparison to previous figures, which was 27% in 2008 [22].  

Considering the C-G equation, the number of CKD patients was overestimated (31%); this was found almost similar with other research findings [22]. One of the main disadvantages of the C-G equation is the requirement of patient’s body weight, which may not always be available in a laboratory set up [23]. On the other hand, the MDRD-4 equation is most familiar among the nephrologists and globally known as a cornerstone method to report CKD [17]. However, the MDRD-4 equation is not without drawbacks as it can offer a false positive approximate when patients present with high eGFR values (Stages 4-5). This study found a reliable percentage of CKD (Stages 3 to 5) after using the CKD-EPI equation, and these findings are consistent with a previous study [24]. Therefore, the CKD-EPI equation might be considered as a better tool to detect the Stage 5 CKD patient when compared with MDRD-4, which supports similar arguments [25]. The CKD-EPI equation is found to be more consistent than the MDRD-4 equation, and it has been found that application of CKD-EPI has increased recently for the clinical settings. However, CKD-EPI still needs validation before starting the use of the routine clinical tests [17].

In our study, according to CKD-EPI equation, the frequency of CKD patients was slightly larger in Stage 5 (0.37%) compared to the MDRD-4 (0.15%). However, there are still controversies to determine which method is better to estimate GFR among the Asians [26]. Our findings are consistent with the previous study where Satirapoj, et al. compared the performance of different methods to identify kidney disease and found bias among the eGFR equations when comparing with direct GFR [27]. Diabetic patients are more vulnerable to develop CKD when compared with non-diabetic patients. A systematic review has shown that the mortality rate of the patients with Stage 3 CKD is higher than those without CKD [28]. Another review has shown that half of the patients suffering from Stage 3 CKD have progressed to Stage 4 and Stage 5 over 10 years [29]. Eventually, these patients will need renal replacement therapy and kidney transplantation in near future. However, the progression of end-stage of CKD can be prolonged by proper detection of eGFR [30].

To the best of researcher’s knowledge, there is no study so far conducted among T2DM patients in Thailand to evaluate the prevalence of kidney disease based on different eGFR equations. This study has found that the CKD-EPI equation can estimate kidney function better than the MDRD-4 and C-G equations, which also corresponds with previous research [27]. Therefore, use of the CKD-EPI equation or a country-specific validated eGFR equation will give the true prevalence of diabetic CKD patients. It will certainly help the policymakers to implement the inclusion of dialysis support services for the true positive CKD patients in Thailand. 

### Limitation of the study

Due to the absence of a gold standard GFR, it was hard to comment which method was more accurate when compared with different equations. Moreover, this study did not compare the performance of the various eGFR equations based on bias, precision, and accuracy.

## Conclusions

eGFR equations can be a suitable method to estimate CKD as a comparison to the conventional serum creatinine measurement. This study found that CKD-EPI equation attempts to overcome the constraint of MDRD-4 and C-G equations to report CKD and might be suitable to use in patients with T2DM.  However, a robust validation of CKD-EPI equation is warranted in Thailand.
